# What does hand motor function tell us about our aging brain in association with WMH?

**DOI:** 10.1007/s40520-020-01683-0

**Published:** 2020-08-29

**Authors:** Misbah Riaz, Torgil Riise Vangberg, Olena Vasylenko, Susana Castro-Chavira, Marta M. Gorecka, Knut Waterloo, Claudia Rodríguez-Aranda

**Affiliations:** 1 Department of Clinical Medicine, UiT the Arctic University of Norway, Tromsö, Norway; 2 PET Center, University Hospital North Norway, Universitetssykehuset Nord-Norge HF, 9038 Tromsö, Norway; 3 Department of Psychology, UiT the Arctic University of Norway, Tromsö, Norway; 4 Department of Neurology, University Hospital of North Norway, Tromsö, Norway

**Keywords:** White matter hyperintensities, Ageing, Pegboard, Finger tapping

## Abstract

**Background:**

White matter hyperintensities (WMH) are a common cerebral finding in older people. WMH are usually asymptomatic, but excessive WMH are associated with cognitive decline and dementia. WMH are also among the neurological findings most consistently associated with declining motor performance in healthy ageing.

**Aims:**

To determine if WMH load is associated with simple and complex motor movements in dominant and non-dominant hands in cognitively intact older subjects.

**Methods:**

Hand motor performance was assessed with the Purdue Pegboard and Finger-tapping tests on 44 healthy right-handed participants, mean age 70.9 years (range 59–84 years). Participants also underwent magnetic resonance (MR) imaging, which were used to quantify WMH volume. The effect of WMH on the motor parameters was assessed via mediation analyses.

**Results:**

WMH load increased significantly with age, while the motor scores decreased significantly with age. WMH load mediated only the relationship between age and left-hand pegboard scores.

**Discussion:**

WMH mediated only the more complex Purdue Pegboard task for the non-dominant hand. This is likely because complex movements in the non-dominant hand recruit a larger cerebral network, which is more vulnerable to WMH.

**Conclusions:**

Complex hand movements in the non-dominant hand are mediated by WMH. Subtle loss of motor movements of non-dominant hand might predict future excessive white matter atrophy.

## Introduction

Late-life motor decline is common in the aging population and is associated with various adverse health outcomes such as gait dysfunction, increased risk of falls, dementia, cognitive decline, and disability [1–3]. Motor decline is multifactorial since it can be caused by neurological or musculoskeletal conditions (e.g., arthritis), or a combination of these factors. These late-life motor deficits interfere with the activities of daily independent living and can lead to functional decline and reduced quality of life in older people.

White matter hyperintensities (WMH) are typically seen as hyperintense lesions in cerebral white matter on fluid-attenuated inversion recovery (FLAIR) MR images and represent atrophy in cerebral white matter [4]. WMH are considered an inevitable consequence of old age, but excessive WMH are seen as an indicator of cerebral small vessel disease [4]. Excessive WMH are also associated with motor impairment and physical disability [5], loss of cognitive functions [6], and increased risk of stroke [7]. Studies find that WMH increase the reaction time on several motor tasks [8, 9], which might explain why WMH are associated with a decline in motor performance, particularly gait and walking speed [10, 11].

Several studies have examined the effect of WMH on both lower [3, 10, 12] and upper extremity motor function [5, 12, 13], but little attention has been paid to whether WMH affect the dominant or non-dominant motor movement the most. Results from fMRI studies suggest that the non-dominant hand may be more vulnerable to WMH than the dominant hand since non-dominant hand movements activate a larger and more distributed brain network than similar movements in the dominant hand [12–14]. Although a functional decline in the non-dominant hand might be less disabling than a similar decline in the dominant hand, it may still degrade the quality of life for those affected. Further, knowledge about how WMH severity relate to motor functioning in the dominant- and non-dominant hands may provide greater insight into the role of WMH in connection with age-related loss of hand motor functioning and could possibly be an early marker of cognitive decline and mobility impairment in the future.

In the present study, we examined how WMH affected simple and complex hand motor function in the dominant and non-dominant hand in cognitively intact elderly participants. Since motor function has been associated with both age and WMH, and WMH is also associated with age [4], we used a mediation model to investigate how WMH might mediate the association between age and upper extremity motor performance.

## Methods

All participants signed written informed consent before participating in the study. The study was approved by the regional ethics committee (REK 2009/1427).

### Participants

The participants were from an ongoing project of motor functions and cognition at the Psychology department of UiT the Arctic University, Norway. Forty-four healthy right-handed elderly people (mean age 70.5 years, 20 females) participated in this study. Participants were screened for cognitive status and depression using the Mini-Mental State Examination (MMSE) [15] and Beck Depression Inventory (BDI) [16], respectively. The participants were recruited via advertisements and flyers at local senior citizens’ centers with the following inclusion criteria: Norwegian as a first language, age greater than 59 years, and right-handed, i.e. a score of 9 or greater on the Briggs–Nebes Handedness Inventory [17]. Exclusion criteria were MRI contraindications, self-reported history of stroke, head trauma, head injury or use of medication known to affect the central nervous system, a diagnosis or pathology that directly affects the musculoskeletal system, recent surgery, acute illness, cardiac/movement disorders, and MMSE < 27. Participants were screened for depression with the Beck Depression Inventory II [18] and none of the participants scored within the depression range. A neuroradiologist screened the MR images and found no major pathologies such as infarctions or tumors.

### Neuropsychological assessment

First, an initial interview was conducted, followed by questionnaires, and the neuropsychological test battery. The neuropsychological assessment for the participants is detailed in [19]. Even though the participants declared themselves as right-handed in the initial interview, the Handedness Questionnaire [17] was used to quantify handedness. The Norwegian version of the SF-36 questionnaire [20] was used to assess physical health.

### Tests of hand motor functions

#### Purdue Pegboard test

The Purdue Pegboard test (PPT) was employed to assess complex hand function in our study. The test apparatus was a board of 22.7 cm × 44.9 cm (Lafayette Instrument Model 32020) with two parallel rows in the middle and four cups at the upper edge. These cups contained pins, washers, collars, and pins from left to right as shown in Fig. 4. The test includes four trials for dominant (right), non-dominant (left), and both hands (simultaneously and alternatively); however, only the first two trails were used in the present study. In the first trial, participants were asked to begin with their dominant (right) hand and to pick up pins from the right-hand cup and insert them into the holes on the right side, starting with the holes farthest from the subject. In the second trial, participants used the non-dominant hand (left) to pick up pins from the left-side cup and insert them into the left column. The pegboard test was modified to accommodate detailed kinematic analysis (used in other parts of the project e.g. [22]). First, the pegboard was colored black, and the pegs and pins were colored red, to ensure adequate contrast between image and the markers attached to the hand. Second, instead of counting inserted pins during a fixed time as in the standard test, participants were asked to put ten pins in the holes (pair of pins in the third task), regardless of the time spent. The reason for modifying the test in this manner was to get an equal number of movement sequences for all participants in the kinematic analysis. The number of trials was ten according to the standardized version of the Purdue Pegboard test [21]. Further details of the procedure are given in [22].

#### Finger tap test

Participants were asked to tap as fast as possible on a lever with the index finger and the lever is attached to a counting device. Finger-tapping score was used in this study because it is a common measure for assessing fine motor control of the upper extremities in a simple motor movement.

#### Grip strength

A hand dynamometer was used to measure grip strength. Since muscular strength declines as a normal process of aging and it is a prerequisite for hand dexterity [23], the grip strength was used to correct for variations in muscle strength in the mediation models.

### MRI scanning

Participants were scanned in a 3 T Siemens Skyra MRI scanner at the University Hospital North Norway. T1-weighted (T1w) images were acquired with a 3D magnetization prepared rapid acquisition gradient-echo (MPRAGE) sequence (flip angle = 9°, TR/TE/TI = 2300 ms/2.98 ms/900 ms). T2-weighted fluid-attenuated inversion recovery (FLAIR) images were acquired with a 3D turbo spin-echo sequence with a variable flip angle (TR/TE/TI = 5000 ms/394 ms/1800 ms, partial Fourier = 6/8). The T1w and FLAIR scans were acquired sagittally with 1 mm in-plane resolution, GRAPPA parallel imaging acceleration factor 2, 80% phase resolution, FOV = 250 mm, 176 slices, 1.2 mm slice thickness, and 256 × 256 image matrix.

### WMH volume measurements

Segmentation of WMH on the FLAIR images was done using the LPA algorithm (https://www.applied-statistics.de/lst.html). Since the accuracy of such algorithms may depend on scanner model and acquisition parameters, the accuracy of the LPA algorithm was validated using an independent sample of 30 subjects (15 males and 15 females aged 61–74 years) from a different study but scanned on the same scanner and with the same image parameters. These images were manually segmented, the gold standard, by M. R. and overseen by T. R. V., and also segmented with the LPA algorithm. The automatic segmentations were visually compared to the manual segmentation and deemed satisfactory. In addition, we computed the Dice score (a measure of the overlap between the manual and automatic segmentation, ranging from 0—no overlap, to 1—perfect overlap). The mean Dice score was 0.6, which is similar to the accuracy of other recent WMH segmentation algorithms [24].

The WMH volume has a known right-skewed distribution [25], which may affect the assumption of normal distribution of the error term and of a linear relationship between the dependent and independent variables for linear regression models. There is also a slight correlation between WMH volume and total brain volume [26], i.e. people with larger brains tend to have slightly larger WMH volume. To make the WMH measure more similar to a normal distribution, and correct for the correlation with the total brain volume, we computed a “WMH load” parameter:

$${\text{WMH}}\,{\text{load}} = \ln \left( {\frac{{{\text{WMH}}\,{\text{volume}}}}{{{\text{ICV}}}}} \right),$$

where WMH is the WMH volume and ICV is the intracranial volume, a measure of premorbid brain volume calculated from the T1w images using a method described in [27]. The WMH load was used in the mediation models instead of the raw WMH volumes.

### Statistical analysis

All statistical analyses were performed in R (ver. 4.0.2) using the “lavaan” package (ver. 0.6) for mediation analyses, “ggplot2” (ver. 3.3.2) for plotting, and the “arsenal” package (ver. 3.5.0) for generating tables of descriptive statistics.

**Fig. 1:**
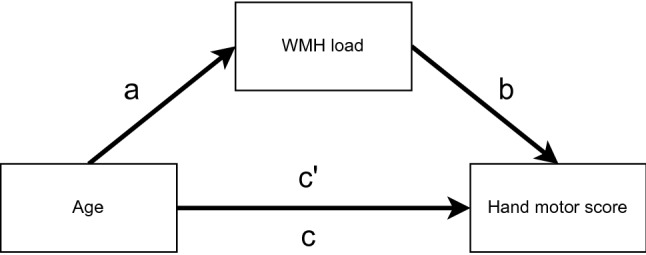
The model used to examine the mediation effect of WMH load on hand motor scores. Here, a is the correlation between age and WMH load, b the relation between WMH load and hand motor score, ab the indirect effect, c is the total effect, and *c*′ the direct effect of age on motor score

We examined the associations between the independent variable (age), the mediator variable (WMH load), and the dependent variables (finger tapping and pegboard test scores) using Pearson correlation. Mediation was used to examine how WMH load might mediate the association between age and hand motor function (Fig. 1). We used the three-step framework for assessing the mediation [28]. The effect of gender, grip strength, smoking, alcohol consumption and body mass index (BMI) was regressed out of this model to avoid confounding by these factors, and the significance of the indirect effect was estimated by bootstrapping with 5000 iterations. There were two missing measurements for BMI, which were replaced by the mean BMI values in the mediation analyses. A *p* value of *p* < 0.05 was considered significant in all analyses.

## Results

### Demographics, motor scores and neuropsychological tests

Descriptive characteristics of the participants in the study are given in Table 1. Summary of the data on motor variables is given in Table 2.

**Table 1:** Summary of participant’s demographics, health and cognitive profile

Demographics and risk factors	*N* = 44
Age [mean (SD), range]	70.5 (5.60), 59–84
Education in years [mean (SD), range]	13.8 (3.50), 7–25
Retired [*N*, (%)]	
Yes	31 (72.1%)
No	12 (27.9%)
Missing	1
Diabetes	
Yes	2 (4.5%)
No	42 (95.5%)
Body mass index (BMI) [mean (SD), range]^a^	26.9 (3.23), 19.4–35.4
Smoking [*N* (%)]	
Never	11 (25.0%)
Previous	28 (63.6%)
Now	5 (11.4%)
Alcohol units pr. week [mean, (SD), range]	4.09 (2.66), 0.25–14.00
MMSE [mean, (SD), range]	29.4 (0.87), 27–30
Trail making test A in seconds [mean, (SD), range]	34.1 (11.27), 18.0–78.0
Trail making test B in seconds [mean, (SD), range]	86.3 (29.51), 42.5–198.0
36-item Short-Form Health Survey [mean, (SD), range]^a^	112.7 (49.13), 85–407
BDI-II [mean, (SD), range]^a^	3.7 (3.40), 0–13
Falls Efficacy Scale International, FES-I [mean, (SD), range]^a^	112.74 (49.13), 85–407
Stroop word [mean, (SD), range]	88.4 (14.04), 47–110
Stroop word–color [mean, (SD), range]	31.52 (7.46), 18–46
Handedness score [mean, (SD), range]^a^	20.00 (4.12), 4–24
Grip strength right hand in kg [mean, (SD), range]	38.5 (10.48), 19.3–60.0
Grip strength left hand in kg [mean, (SD), range]	37.0 (10.44), 18.3–64.3

^a^BMI: 2 missing, 36 items: 5 missing, BDI-II: 2 missing, FES-I: 2 missing, handedness score: 2 missing

### White matter hyperintensities

**Fig. 2:**
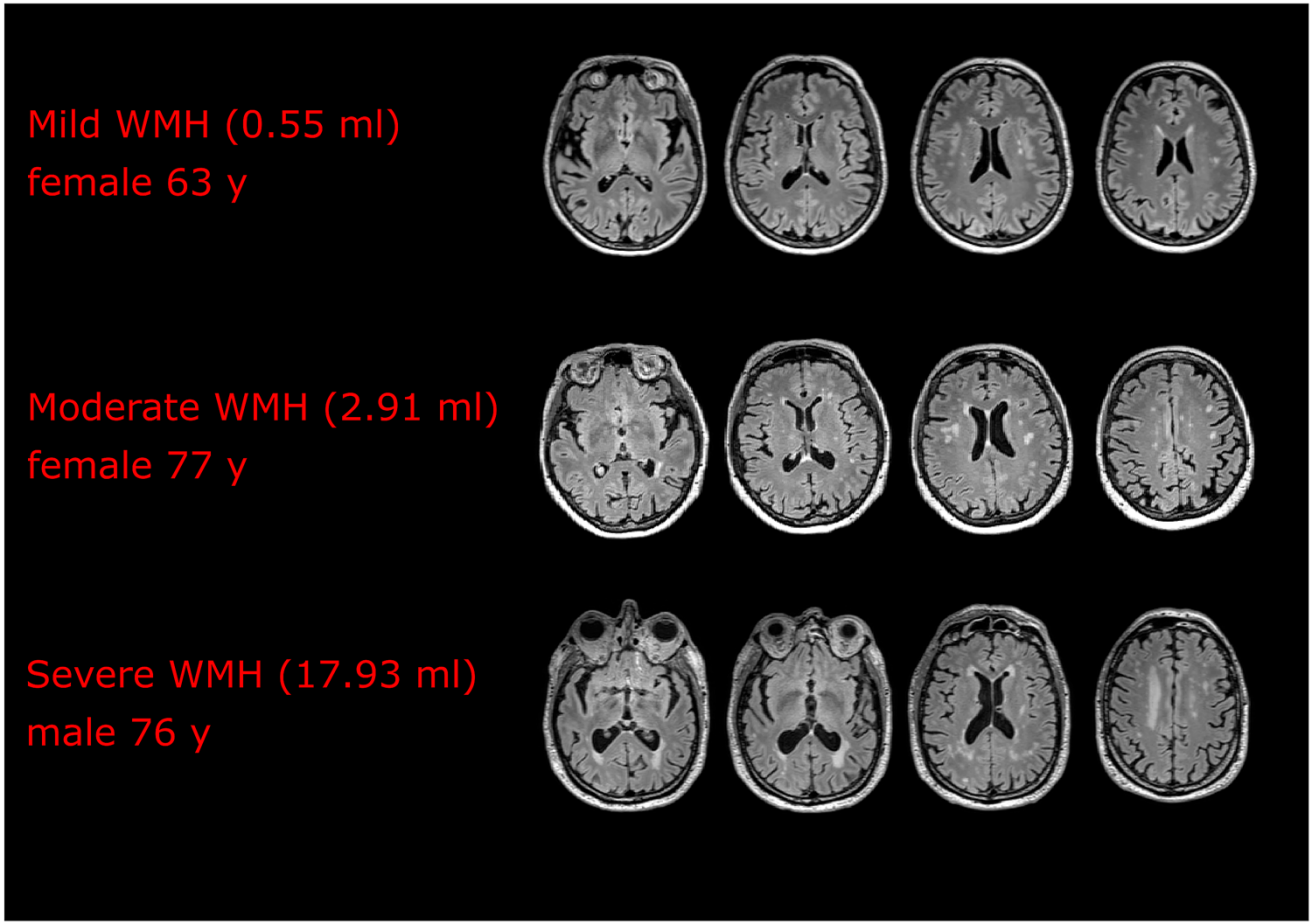
WMH volume for three participants with differing volumes ranging from mild to severe (four slices for each subject are shown)

WMH were present in all our subjects, but their extent and distribution varied considerably. The median WMH volume was 0.97 ml, range 0.05–17.93 ml (mean 2.32 ml, SD = 3.34) (Table 2). The varying severity of WMH in the participants is illustrated in Fig. 2, with an example of mild (0.55 ml), moderate (2.91 ml), and severe (17.93 ml) WMH.

**Table 2:** Summary of the data on motor performance and WMH load

	Total (*N* = 44)	
	Mean (SD)	Range
Pegboard right hand (s)	26.31 (5.48)	17.02–38.78
Pegboard left hand (s)	27.14 (4.73)	17.44–38.72
Finger tapping right hand (number of taps)	41.23 (8.77)	20.33–54.67
Finger tapping left hand (number of taps)	38.17 (7.96)	20.33–55.33
WMH load	− 7.30 (1.40)	− 10.34 to − 4.54

### Correlation analyses

**Fig. 3:**
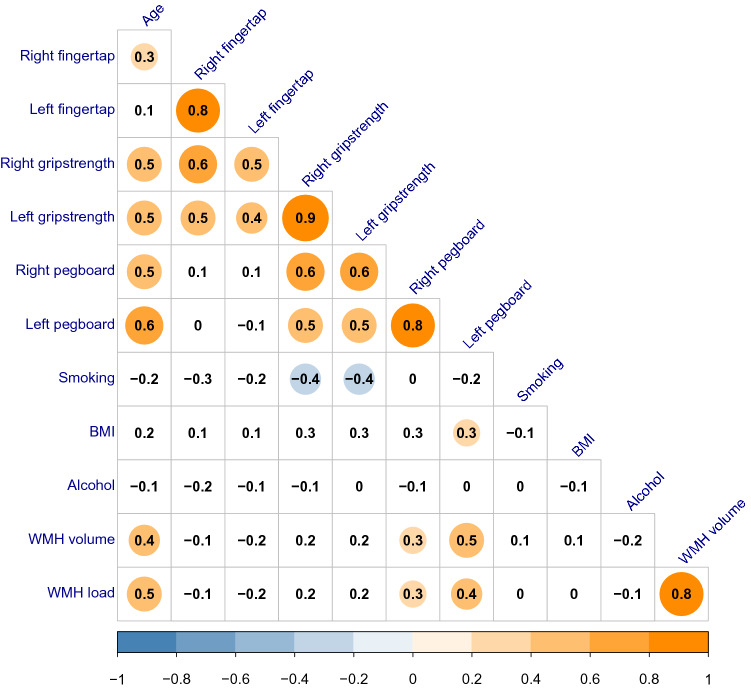
Plot showing correlations between grip strength, motor scores, age, smoking, BMI, alcohol consumption and WMH load. The numbers in each box are Pearson’s *r*, and the circles indicate significant correlations (*p* < 0.05)

From the pairwise correlations (Fig. 3), we observed that the pegboard scores were significantly correlated with age (right hand: *r* = 0.49, *p* = 0.0008, left hand: *r* = 0.55, *p* = 0.0001) and WMH load (right hand: *r* = 0.30, *p* = 0.048, left hand: *r* = 0.44, *p* = 0.003). The finger tapping scores were only significantly correlated with age for the right hand (*r* = 0.31, *p* = 0.0411), demonstrating that the finger tapping scores were less associated with both age and WMH compared to the pegboard scores.

### Mediation analysis

The results from the mediation analyses are summarized in Table 3. For finger tapping scores, the associations between age and the right and the left-hand finger tapping scores, i.e. the total effect *c*, were not significant (*β* = − 0.03, *p* = 0.9 and *β* = − 0.23, *p* = 0.2 respectively), thus excluding a mediating effect of WMH.

**Table 3:** Mediation effect of WMH load on the association between age and motor scores (standardized regression coefficients)

	Path *a*	Path *b*	Indirect effect ‘*ab*’	Total effect (*c*)	Direct effect (*c*′)
Pegboard right hand	0.56**	0.17	0.09	0.15	0.06
Pegboard left hand	0.57**	0.31*	0.18*	0.35*	0.17
Finger tap right hand	0.56**	− 0.23	− 0.13	− 0.03	0.10
Finger tap left hand	0.57**	− 0.30	− 0.17	− 0.23	− 0.06

Values for paths *a* and *b* represent the association between age and WMH load, and the associations between the WMH load and motor scores respectively. Path *c* is the total effect of age on motor scores, and path *c*′ the direct effect of age on motor scores after controlling for the WMH load as a mediating variable

*p* < 0.05*; *p* < 0.01**; *p* < 0.001***

**Fig. 4:**
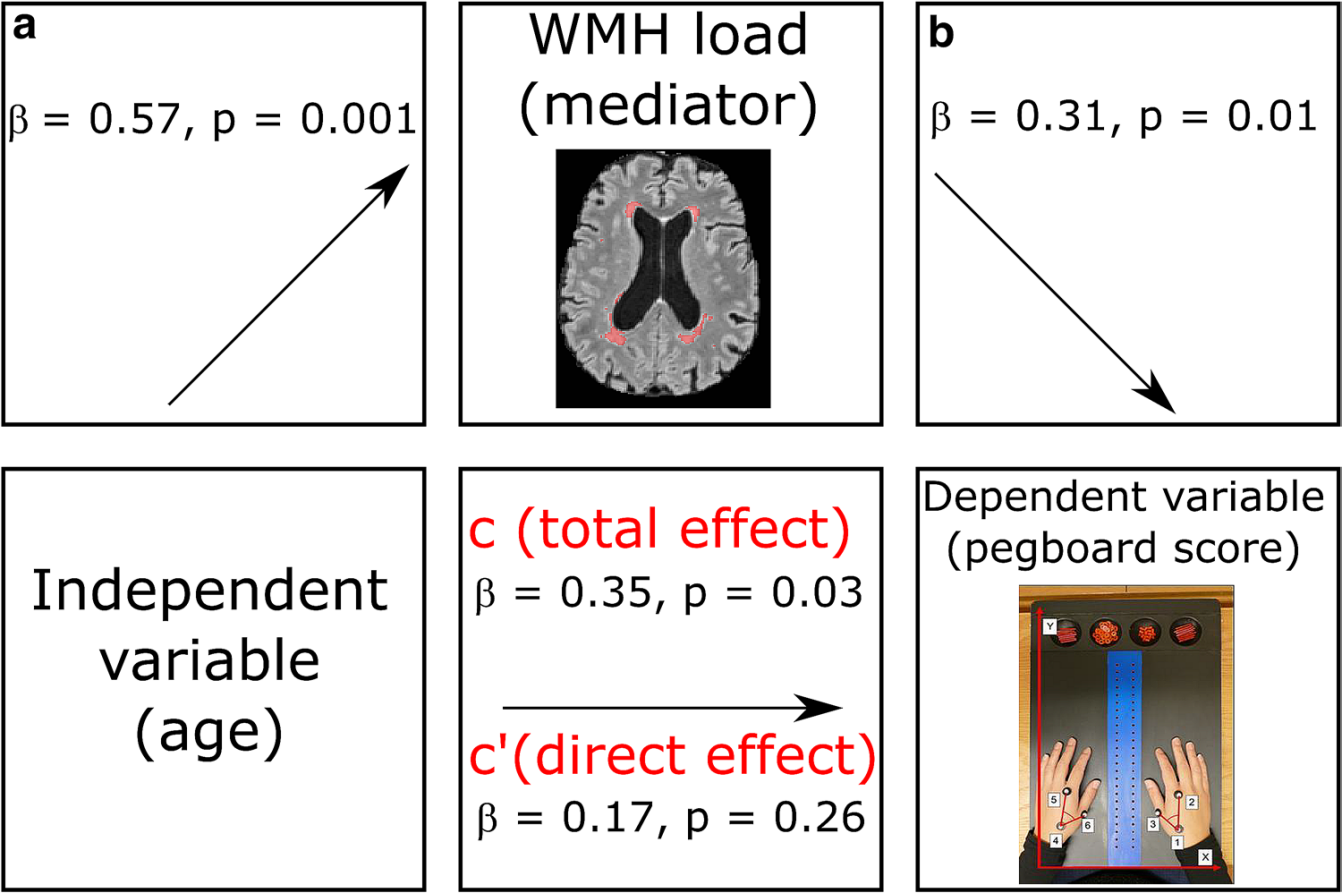
Mediation analyses—values for paths *a* and *b* represent the association between age and the WMH load, and the association between the WMH load and left-hand pegboard scores respectively. Path *c* shows the total effect of age on left-hand pegboard scores

For the mediation analysis of the right-hand pegboard scores, the total effect was not significant (*β* = 0.15, *p* = 0.24), and hence no mediation either. However, the association between age and left-hand pegboard scores was significant (*β* = 0.35, *p* = 0.03), which was fully mediated by WMH load (*β* = 0.18, *p* = 0.02). As shown in path diagram, the regression coefficient between age and WMH load was statistically significant (*β* = 0.57, *p* = 0.001), as was the regression coefficient between WMH load and left-hand pegboard scores (*β* = 0.31, *p* = 0.01), as illustrated in Fig. 4. The indirect effect was also statistically significant (*β* = 0.18, *p* = 0.02).

## Discussion

The results show that independent of age, gender, grip strength, and WMH risk factors, WMH load mediated the hand motor performance in the non-dominant hand, but only for complex motor movements. These findings are consistent with previous studies showing that WMH are related to poorer motor function [5, 29, 30]. To our knowledge, however, previous studies have not examined whether WMH are associated with complex motor movements differently than simple movements, or whether WMH correlates preferentially with the dominant or non-dominant hands.

Even though pegboard scores of the right and the left hand were highly correlated (*r* = 0.8, Fig. 3), WMH had a mediating effect only on the left-hand. Previous research suggests that non-dominant motor activity depends on a more distributed functional brain network, with inter-hemispheric and cortical—cerebellar communication compared to the movement of the dominant hand [12, 31, 32]. Since WMH disrupt the signaling in cerebral white matter leading to cortical disconnection [33, 34], it is likely that a more extensive functional network (left-hand motor functioning) is more vulnerable to WMH than a more localized network (right-hand motor functioning). The reason WMH only mediated the complex hand motor movement, can be explained by a similar argument since complex hand movements activate a more extensive bilateral functional network compared to simple hand movements [12, 31].

Our findings suggest that WMH strongly correlate with complex motor movements of the non-dominant hand in the absence of any functional decline in the dominant hand. Hence, the motor changes in the non-dominant hand in apparently healthy elderly should be further assessed as to whether these changes predict major motor loss or pathological cognitive impairment. We also note that of the cognitive tests, the TMT A and Stroop word-color tests showed the strongest associations with WMH load (*r* = 0.48, *p* = 0.001 and *r* = − 0.29, *p* = 0.057, respectively), in agreement with previous studies [34–37], and showed also the strongest associations with left-hand pegboard scores (*r* = 0.52, *p* = 3e−4 and *r* = − 0.56, *p* = 9e−5). This suggests that the left-hand pegboard test, the TMT A and Stroop word–color are particularly sensitive to WMH load. In a clinical setting, administering these three tests might be a sensitive predictor of subclinical WMH and future motor and cognitive decline. However, future longitudinal studies are necessary to corroborate the present data, as our cross-sectional study cannot prove such causal connection.

A notable limitation of the present study is the cross-sectional design making it impossible to determine causal effects. In addition, a relatively small sample was evaluated (*n* = 44) and the age range for the participants was wide (59–84 years). The interpretation of our results is further limited to healthy, cognitively intact, community-dwelling older people, and the findings might be different for subjects with cognitive decline for example.

In conclusion, WMH load mediated only the pegboard scores of non-dominant (left) hand in a healthy cognitively intact elderly cohort. Complex motor movements in the non-dominant hand likely require a more extensive cerebral network, than the other tasks considered, which in turn might be more susceptible to interference by WMH. The results also suggest that complex motor movements of the non-dominant hand might be a predictive marker for excessive WMH.

## Availability of data and material

The ethics approval does not allow us to share the raw data, but upon reasonable request, summary statistics can be obtained from the corresponding author.
